# Chromosome-level genome of *Thymus mandschuricus* reveals molecular mechanism of aroma compounds biosynthesis

**DOI:** 10.3389/fpls.2024.1368869

**Published:** 2024-03-13

**Authors:** Lin Jia, Ning Xu, Bin Xia, Wenjie Gao, Qingran Meng, Qiang Li, Ying Sun, Shoubin Xu, Miao He, Huiyan Gu

**Affiliations:** ^1^ School of Forestry, Northeast Forestry University, Harbin, China; ^2^ College of Landscape Architecture, Northeast Forestry University, Harbin, China; ^3^ School of Ecological Technology and Engineering, Shanghai Institute of Technology, Shanghai, China; ^4^ School of Perfume and Aroma Technology, Shanghai Institute of Technology, Shanghai, China; ^5^ Heilongjiang Academy of Forestry, Harbin, China

**Keywords:** *Thymus mandschuricus*, phylogeny, CYP450, terpenoid biosynthesis, aroma production

## Abstract

**Background:**

*Thymus mandschuricus* is an aromatic and medicinal plant with notable antibacterial and antioxidant properties. However, traditional breeding methods rely on phenotypic selection due to a lack of molecular resources. A high-quality reference genome is crucial for marker-assisted breeding, genome editing, and molecular genetics.

**Results:**

We utilized PacBio and Hi-C technologies to generate a high-quality chromosome-level reference genome for *T. mandschuricus*, with a size of 587.05 Mb and an N50 contig size of 8.41 Mb. The assembled genome contained 29,343 predicted protein-coding genes, and evidence of two distinct whole-genome duplications in *T. mandschuricus* was discovered. Comparative genomic analysis revealed rapid evolution of genes involved in phenylpropanoid biosynthesis and the CYP450 gene family in *T. mandschuricus*. Additionally, we reconstructed the gene families of terpenoid biosynthesis structural genes, such as TPS, BAHD, and CYP, and identified regulatory networks controlling the expression of aroma-synthesis genes by integrating transcriptome data from various organs and developmental stages. We discovered that hormones and transcription factors may collaborate in controlling aroma-synthesis gene expression.

**Conclusion:**

This study provides the first high-quality genome sequence and gene annotation for *T. mandschuricus*, an indigenous thyme species unique to China. The genome assembly and the comprehension of the genetic basis of fragrance synthesis acquired from this research could potentially serve as targets for future breeding programs and functional studies.

## Background

The family Labiatae is among the biggest in flowering plants, containing 236 genera and over 7,000 species (Raja, 2012). With its attractive appearance, special taste, strong scent, and therapeutic capability, *Labiatae* has significant ecological, economic, and cultural importance due to rich phytochemical compositions (Mulas, 2006). Because of these desirable qualities, it is extensively cultivated worldwide. *T. mandschuricus*, a member of the Lamiaceae family, is widely used as a spice and a traditional Chinese herb (Saroukolai et al., 2010; Kim et al., 2003; Qiao et al., 2021; Koul et al., 2008). It is often found in the northern part of China and can be applied in many occasions: traditional Chinese medicine, household cleaning, environmental safeguarding, and even in the preparation of mutton dishes. The extract of *T. mandschuricus* has been shown to have anti-cancer, anti-bacterial, and anti-inflammatory properties (Afonso et al., 2020; Gordo et al., 2012).

Natural antifungal agents from plant essential oils and their active components are a hot area. *T. mandschuricus* has strong floral and leaf aromas from which aromatic oils were extracted in industry and usually used for medical purposes (Wang et al., 2018). Previous studies have shown that thymol and carvacrol are the main components of essential oils in *T. mandschuricus* and have good antifungal activity (Hosseinzadeh et al., 2015). Bioactive components such as thymol, carvacrol, citral, geraniol, and nerolidol are responsible for the therapeutic benefits of *T. mandschuricus* as antibacterial and antioxidant agent (Nieto, 2020; Tohidi et al., 2017; Martin et al., 2003). Most of these substances belong to the terpenoid family. Examining terpene synthases provides an excellent opportunity to gain insights into the evolutionary aspects of terpenoid biosynthesis. Building blocks for terpenoids in plants come from either the cytosolic mevalonate (MVA) or plastidial methylerythritol phosphate (MEP) pathways (Tholl, 2006; Dudareva and Pichersky, 2008; Chen et al., 2020) and include isopentenyl diphosphate (C-5) and isomeric dimethylallyl diphosphate. The enzymes cytochrome P450 (CYP450), acyltransferases, 2-oxoglutarate-dependent dioxygenases, methyltransferases, and glycosyltransferases change and further diversify scaffolds, making terpenoids structurally varied.

It seems that the evolution of plant gene clusters, which resulted in the diversity of specialized terpenoids, was driven by gene duplication and neofunctionalization (Godden et al., 2019; Panchy et al., 2016). Gene duplication is common in plant genomes, with the most common mechanisms being whole-genome duplication (WGD) and tandem duplication (Chen et al., 2011). A number of plant families, including TPS (trehalose phosphate phosphatase), CYP450, and BAHD (BAHD family of acyltransferases), expand their gene repertoires through both mechanisms (Nelson and Werck-Reichhart, 2011; Weitzel and Simonsen, 2015; Stracke et al., 2020). These repetitions give useful material that may assist in the evolution of various activities, including pest resistance and stress tolerance, all of which improve plant adaptation (Xu et al., 2021; Hansen et al., 2017). There may be less chance of one-way gene loss due to recombination events and inadequate cluster expression if genes involved in terpenoid biosynthesis are clustered together. The diversification of monoterpenes and sesquiterpenes is intimately linked to the growth of the terpenoid biosynthetic gene family. Increases in the size of the TPS-b subfamily are positively connected with increases in the variety of monoterpenes found in plants (Jones et al., 2011), whereas the TPS-a subfamily is responsible for the synthesis of sesquiterpenes (Kejnovsky et al., 2009).

Using PacBio sequencing and chromatin conformation capture technologies, we have determined the reference genome sequence of *T. mandschuricus*. Mechanisms for polyphenol and terpenoid production in *T. mandschuricus* have been uncovered using comparative genomic, phylogenetic, and transcriptomic analyses as well as genome-scale sequencing data processing. This species is presumed to be haploid with a chromosome number of n = 13 (Sun et al., 2022). This study offers new insights into molecular breeding and the identification of functional genes associated with key traits of thyme. Moreover, the *T. mandschuricus* genome presented herein serves as a valuable resource for future investigations.

## Results

### Chromosome-level genome assembly and annotation of *T. mandschuricus*

**Figure 1:**
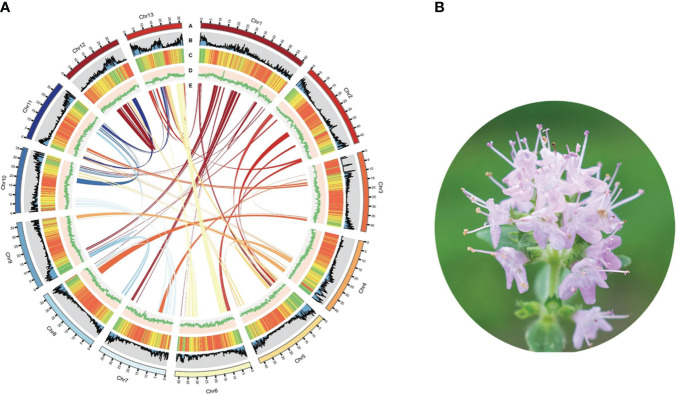
Summary of *T. mandschuricus* genome assembly. **(A)** Circos plot showing *T. mandschuricus* genomic features: A, karyotyping results; B, protein-coding gene density; C, transposable element density; D, GC content; and E schematic presentation of major interchromosomal relationships in the *T. mandschuricus* genome. **(B)** Photo of *T. mandschuricus* in blooming period (FS).

Utilizing PacBio and Illumina HiSeq sequencing technologies, we established the complete genome sequence of *T. mandschuricus*. PacBio third-generation sequencing yielded 23 Gb (43.50×) of clean data, whereas sequences from Illumina sequencing were 117.26× in depth. Sequence reads were collected, trimmed, removed of contaminated data, and finally assembled. The assembled *T. mandschuricus* genome spans 587.05 Mb and contains a contig N50 of 8.41 Mb, which was not far from the k-mer–based estimate. The mounting rates of *T. mandschuricus* scaffold assembly on the 13 chromosomes using HiC assembly were all more than 90% (**Figure 1A**; **Supplmentary Table S1**). Short reads aligned to the reference genome was at a rate of above 98%. With an examination of 303 conserved core genes, BUSCO demonstrated that the *T. mandschuricus* genome was more than 96.8% complete; with an evaluation of 458 conserved core genes of eukaryotes, CEGMA demonstrated that the *T. mandschuricus* genome was more than 95.56% complete. These results indicate that our genome assembly is of high quality. The genome guanine and cytosine (GC) ratio, GC skew, unknown bases (N), and gene density for each chromosome were also calculated, and the findings showed that various chromosomes had significantly diverged in some cases (**Figure 1A**). We predicted 29,343 protein-coding genes, 4,474 microRNAs, 2,198 rRNAs, 856 transfer RNAs (tRNAs), and 2,106 small nuclear ribonucleic acid (snRNAs) in the assembled genome. According to the results of comprehensive functional annotation of all genes, 28,205 out of 29,343 have established roles in at least one database.

Furthermore, we employed structure prediction and *de novo* prediction to build a database of the genome’s repeat sequences. According to the findings, 637,535 repeat sequences account for 67.70% of the whole genome in *T. mandschuricus*. The three most prevalent forms of repetition in the genome are trithoracic repeat elements (TRF), long terminal repeats (LTR), and long interspersed nuclear elements (LINE). The distribution of major repeats was analyzed throughout the genome, and the results showed that they dispersed differently depending on chromosome and location (**Figure 1A**). In addition, we compared the *T. mandschuricus* genome with that of *Salvia splendens* to look for collinear regions (**Supplementary Figure S1**). By comparison, 4,495, 30, and 2,949 loci were found for chromosomes 1, 2, and 6, respectively. *T. mandschuricus* and *S. splendens* share many genetic similarities. However, the fact that orthologous sites in *T. mandschuricus* are dispersed over many chromosomes of *S. splendens* suggests that structural variation and replication timing have been distinct between the two species (**Figure 1B**). More gene cluster duplications and possibly entire genome duplications may have occurred in *S. splendens*.

### The phylogeny and evolution of *T. mandschuricus*

**Figure 2:**
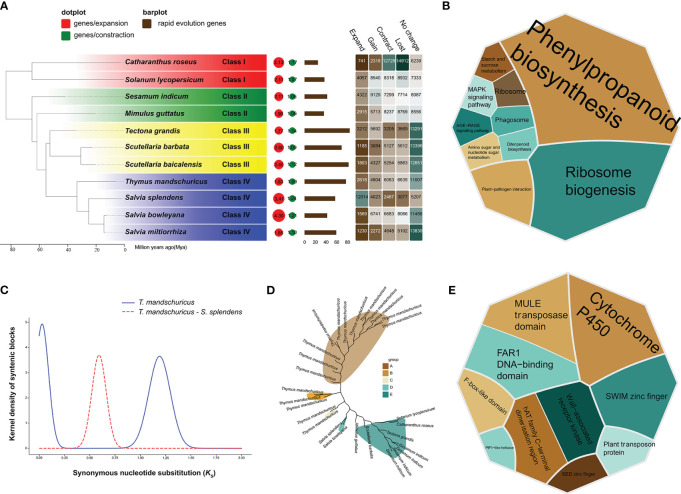
Phylogenetic study of T. mandschuricus with 10 distinct species, including Salvia splendens, Salvia bowleyana, Salvia miltiorrhiza, Mimulus guttatus, Sesamum indicum, Catharanthus roseus, Solanum lycopersicum, Tectona grandis, Scutellaria barbata, and Scutellaria baicalensis. **(A)** A phylogenetic tree of 11 different species based on single-copy genes. Bar plot indicates the number of genes in each species that have undergone fast evolution, and heatmap reflects the number of genes that have undergone expansion, contraction, loss, or gain in each species, respectively. Circles symbolize recent whole-genome duplication (WGD) occurrences. **(B)** A KEGG functional enrichment study of genes expanded gene family in T. mandschuricus. **(C)** An analysis of WGD events that have taken place in T. mandschuricus and other species. **(D)** Expansion of the gene family OG0002816 related to phenylpropanoid metabolism in T. mandschuricus. **(E)** PFAM clustering of genes in T. mandschuricus that have undergone fast evolutionary change.

We were able to compare the *de novo*–assembled *T. mandschuricus* genome with those of the 10 previously described species by employing protein-coding genes from all of these plants. To investigate the evolutionary history of *T. mandschuricus*, we analyzed the genomes of 11 selected angiosperm species. There were many variations in the number of copies of gene families between species, and a total of 69 single-copy orthologous genes were found in eleven different types of organisms (**Figure 2A**). Our findings suggest that *T. mandschuricus*, along with other members of the family Lamiaceae—*Salvia splendens*, *Salvia bowleyana*, and *Salvia miltiorrhiza*—all belong to the same clade. The estimated divergence time showed that *T. mandschuricus*, *Salvia splendens*, *Salvia bowleyana*, and *Salvia miltiorrhiza* may have gradually diverged from their most recent common ancestor (MRCA) around the same period at 53.21 million years ago (Mya) (**Figure 2A**). We examined the reduction and growth of 11 gene families in *T. mandschuricus* to better understand how this species adapts to the environment. A total of 6,635 genes were lost in *T. mandschuricus*, whereas 6,083 shrank in *T. mandschuricus* (from 15 gene families). Fast evolution occurred in 600 of these gene families in *T. mandschuricus* (**Figure 2A**). Analysis of the expanded gene families in *T. mandschuricus* revealed functional similarities to the phenylpropanoid biosynthesis, diterpenoid biosynthesis, MAPK:Mitogen-Activated Protein Kinase (MAPK) signaling pathway, ribosome biogenesis, and other pathways (**Figure 2B**). While this went on, *T. mandschuricus* may have experienced two distinct WGDs: one around 69.5 Mya and the other at 3.49 Mya (**Figures 2A, C**). There was a massive increase in the number of genes in the *T. mandschuricus* family, where OG0002816 was shown to be involved in phenylpropanoid biosynthesis (**Figure 2D**). One of the genetic processes of *T. mandschuricus* to create distinctive fragrances may be the metabolism of phenylpropanoid and diterpenoid, which is linked to the formation of polyphenols, terpenoids, and other aromas. Furthermore, PFAM clustering of rapid evolving genes in *T. mandschuricus* revealed that they are mostly associated with cytochrome P450, MULE transposase domain, and other activities (**Figure 2E**); cytochrome P450 genes (CYP) may be involved in the manufacture of terpenoids in *T. mandschuricus.*

### Pathway and structural gene reconstruction of polyphenol synthesis

**Figure 3:**
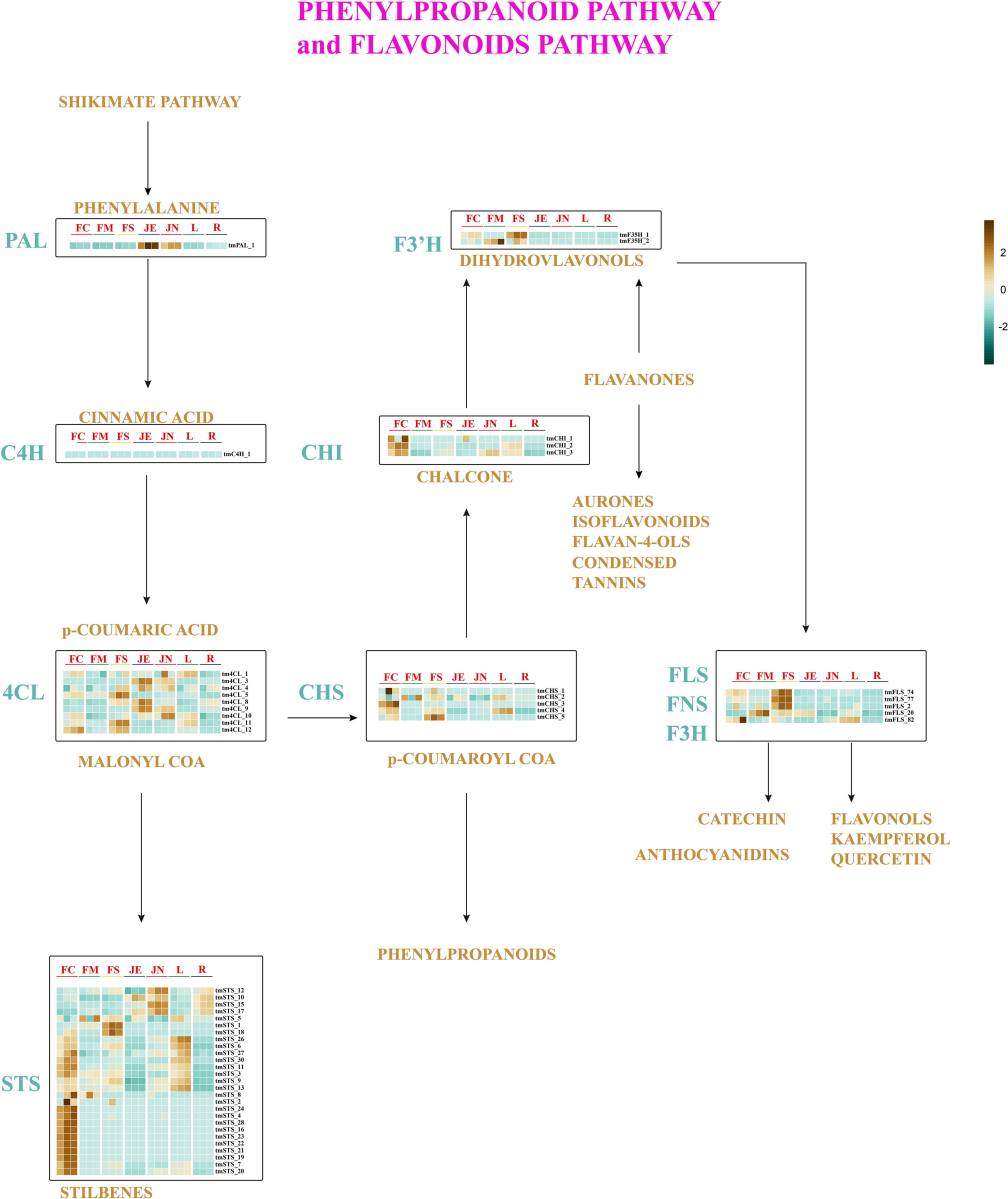
Pathways for phenylpropane metabolism and flavonoids metabolism in *T. mandschuricus*. Abbreviations for these enzymes are as follows: PAL, phenylalanine ammonia lyase; C4H, cinnamate-4-hydroxylase; 4CL, coumarate CoA ligase; STS, stilbene synthase; CHS, chalcone synthase; CHI, chalcone isomerase; FNS, flavone synthase; F3H, flavanone 3-hydroxylase; F3′H, flavonoid 3′ hydroxylase; F3′5′H, flavonoid 3′5′ hydroxylase; FLS, flavonol synthase; FC, initial flowering stage; FM, final flowering stage; FS, blooming period; JE, secondary branch; JN, tender stem; L, leaf; R, root.

Thymol, a phenolic chemical, significantly contributes to the distinctive odor of *T. mandschuricus*. Previous studies have demonstrated that thyme possesses a wide array of phenolic and monoterpenoid compounds, owing to its rich volatile component content. Analysis of the genomes of *T. mandschuricus* and 10 other species revealed a notable expansion in the number of gene families associated with phenylpropanoid production. The shikimate pathway generates shikimic acid from the combination of phosphoenolpyruvate produced during glycolysis and erythrose-4-phosphate generated during the pentose phosphate cycle. L-phenylalanine, along with other aromatic amino acids, is created by the second route. We have identified an l-phenylalanine-dependent polyphenol production pathway (**Figure 3**). Structural genes such as PAL (phenylalanine ammonia lyase), C4H (cinnamate-4-hydroxylase), 4CL (coumarate CoA ligase), STS (stilbene synthase), CHS (chalcone synthase), CHI (chalcone isomerase), FNS (flavone synthase), F3H (flavanone 3-hydroxylase), and F3′H (BnF3′H-1) were identified (**Figure 3**).

To further elucidate the pathways involved in phenylpropane metabolism and flavonoid metabolism in *T. mandschuricus*, we analyzed RNA-seq data from the genome. All three tmCHI genes show significantly upregulated at initial flowering stage (FC). Conversely, 22 tmSTS genes, including tmSTS 9 and tmSTS 13, were significantly downregulated at secondary stem (JE) and root (R) (**Figure 3**). A number of tmSTS, tmF3′5′H, tmFLS, tmF3H, and tmFNS were identified to be uniquely expressed at different phases of floral development, suggesting a robust synthesis of diverse polyphenols throughout the flower development of *T. mandschuricus*. The specific expression of FLS (FNS and F3H) in flowers indicates that a significant portion of phenylpropanes in *T. mandschuricus* flowers is synthesized during the floral development stage. The key genes involved in flavonoid biosynthesis such as STS, CHS, and CHI are expressed in flowers, stems, and leaves of *T. mandschuricus*, providing molecular insights into the fragrance in stems and leaves of *T. mandschuricus*.

### Identification and phylogeny of terpenoid biosynthesis-related genes

**Figure 4:**
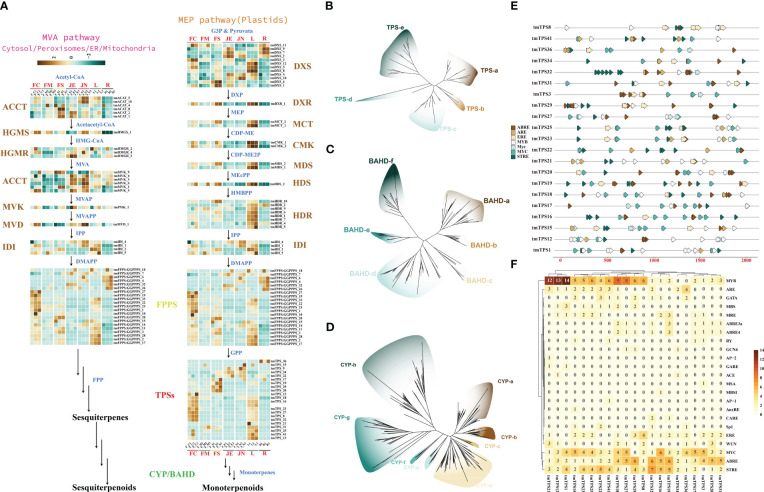
Reconstruction and phylogeny of the terpenoid biosynthesis pathway in *T. mandschuricus.* **(A)** Pathways of terpene biosynthesis in *T. mandschuricus* and relative expression of structural genes therein. These include acyl-coenzyme A:cholesterol acyltransferase (ACAT), hydroxymethylglutaryl coenzyme A synthase (HMGS), hydroxymethylglutaryl coenzyme A reductase (HMGR), mevalonate kinase (MVK), phospho-mevalonate kinase (PMK), mevalonate diphosphate decarboxylase (MVD), 1-deoxy-D-xylulose 5-phosphate synthase (DXS), 1-deoxy-D-xylulose 5-phosphate reductoisomerase (DXR), 2-C-methyl-D-erythritol-4-phosphate cytidylyltransferase (MCT), 4-(cytidine-5-diphospho)-2-C-methyl-D-erythritol kinase (CMK), 2-C-methyl-D-erythritol-2,4-cyclodiphosphate synthase (MDS), **(E)**-4-hydroxy-3-methyl-but-2- enyl-pyrophosphate synthase (HDS), **(E)**-4-hydroxy-3-methyl-but-2-enyl-pyrophosphate reductase (HDR), isopentenyl diphosphate isomerase (IDI), and lavandulyl diphosphate synthase (LPPS). **(B–D)** Phylogenetic analysis of TPS, BAHD, and CYP gene families in *T. mandschuricus*, respectively. **(E)** The primary transcriptional regulatory components in the upstream 2k region of the TPS gene promoter are shown here in the form of a schematic diagram. **(F)** Prediction of transcription factor node sites in the upstream 2k region of the promoter of the TPS gene expressed in flower development of *T. mandschuricus*.

Analysis of the genomes of *T. mandschuricus* and 10 other species showed that the gene family responsible for diterpenoid synthesis had significantly expanded. From the 2-C-methyl-D-erythritol-4-phosphate (MEP) and mevalonate (MVA) routes, two 5-carbon “building blocks” [isopentenyl diphosphate (IPP) and dimethylallyl diphosphate (DMAPP)] are produced, which are then used in the biosynthesis pathway of terpenoids in plants. Members of 14 gene families are expressed in the terpene synthesis pathway. These includes acyl-coenzyme A:cholesterol acyltransferase (ACAT), hydroxymethylglutaryl coenzyme A synthase (HMGS), hydroxymethylglutaryl coenzyme A reductase (HMGR), mevalonate kinase (MVK), and phospho-mevalonate kinase (PMK). (**Figure 4A**). tmCMK 1 and tmCMK 2 exhibited a significant upregulation in the leaf (L), whereas they demonstrated a significant downregulation in the root (R) (**Figure 4A**). The MEP and MVA pathways were identified by transcriptome analysis. Terpene production in *T. mandschuricus* may include multi-organ cooperation and transport. Genes involved in the first half of the process (structural genes) were significantly expressed in leaves and secondary stems, whereas genes involved in the second half (FPPS/GPPS) were substantially expressed in flowers. The GPP and FPP reactions, catalyzed by tmTPSs, formed the C10 backbone of monoterpenes and C15 backbone of sesquiterpenes, respectively. We found 47 TPS-encoding genes that fell into five distinct groupings based on common conserved patterns (**Figures 4B-D**). During the blossoming and development of *T. mandschuricus*, we discovered 21 highly expressed tmTPSs in the flowers. The cytochrome P450 (CYP450) enzyme is responsible for the hydroxylation of monoterpenes and sesquiterpenes, whereas the BAHD family of plant acyltransferases is responsible for the esterification of these compounds. We found 338 CYP450 and 138 BAHD gene family members, approximately the same numbers as other types of spices. In addition, we found that genes in the terpenoid-synthesizing CYP450 and BAHD families are expressed at precise times throughout flower development, suggesting their importance in *T. mandschuricus*. We also examined the regulatory elements of transcription factors in the upstream area of the flower-expressed tmTPS promoter and found a high concentration of V-myb myeloblastosis viral oncogene 245 homolog (MYB), Myelocytomatosis transcription factors (MYC), antioxidant 246 responsive element (ARE), estrogen response element (ERE), ABA-responsive element (ABRE), stress response element (STRE) and other regulatory elements (**Figures 4E, F**). This may suggest that environmental influences, hormones, and endogenous transcription factors all play roles in regulating tmTPS gene transcription.

### Regulatory analysis of tmTPS genes

**Figure 5:**
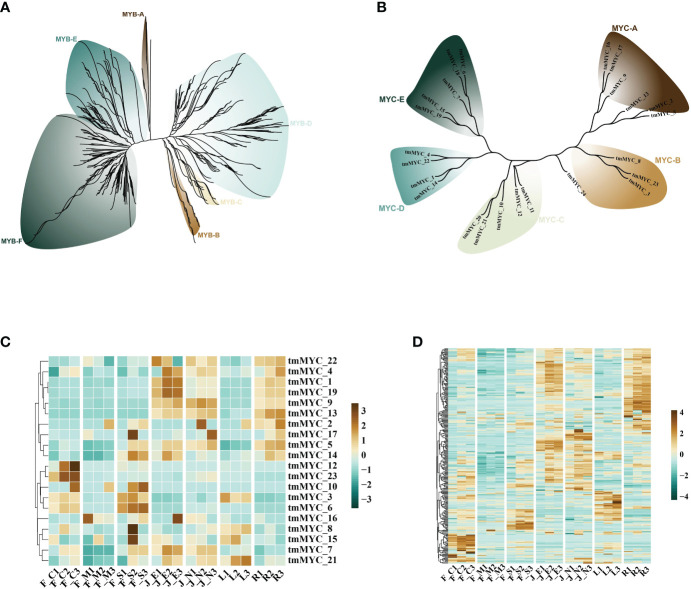
Analysis of regulatory systems governing the expression of TPS genes during flower development in *T. mandschuricus*. **(A, B)** Phylogenetic analyses of the MYB and MYC transcription factor families, respectively, in *T. mandschuricus.* **(C, D)** Relative expression levels of genes belonging to the MYB and MYC transcription factor families in *T. mandschuricus* during various stages of flower development, respectively. FC, initial flowering stage; FM, initial flowering stage; FS, blooming period; JE, secondary branch; JN, tender stem; L, tender stem; R, root.

Terpene synthase genes (TPSs) are critical structural genes that regulate terpene synthesis in *T. mandschuricus*, because they catalyze the synthesis of the essential framework for monoterpenes (C10) and sesquiterpenes (C15). Researchers found a plethora of gene regulatory elements, including MYB, MYC, ARE, ERE, ABRE, and STRE, in the promoter region of the tmTPS gene (**Figure 4E**). MYB binding sites were abundant in the upstream 2k regions of the promoters of many genes, including tmTPS8, tmTPS41, tmTPS32, tmTPS1, tmTPS12, and tmTPS21; other MYB binding genes were identified as tmMYB 11, tmMYB 184, tmMYB 290, and tmMYB 151. This may indicate that transcription of TPS genes is under the control of MYB transcription factors. Numerous MYB and TPS genes were expressed at the same time during the first stage of flower development in *T. mandschuricus*, and these MYB transcription factors were classified into five distinct subclasses (**Figures 5A, C**). *T. mandschuricus*’s MYC transcription factor family is likewise split into five groups, with subgroups B and C being particularly abundant in floral tissues (**Figures 5B, D**).

STRE elements concentrated in the upstream 2k areas of the promoters of genes including tmTPS32, tmTPS25, tmTPS22, tmTPS19, and tmTPS16, suggesting that the transcription of these genes may be activated by biotic and abiotic stimuli. Another putative regulatory link between TPS and MYC is suggested by the high expression of the MYC transcription factor family members (tmMYC12, tmMYC23, and tmMYC10) in *T. mandschuricus* flowers (**Figure 5D**).

## Discussion

Plants genomes are different from those of animals in that they are more variable, complex, and redundant (Murat et al., 2010). Plants are particularly vulnerable to environmental stress because of their immobile state (Tank et al., 2015). It is believed that genomic redundancy acts as raw materials for plant species diversification and evolutionary novelty via polyploidy/WGD events, which are often accompanied by a large number of repetitive genes, significant genome rearrangements, and increases in genetic variety (Wang et al., 2020; Jia et al., 2021). A calculation of the divergence period reveals that *T. mandschuricus*, *Salvia splendens*, *Salvia bowleyana*, and *Salvia miltiorrhiza* may have progressively diverged at 53.21 Mya from their MRCA. As time goes on, *T. mandschuricus* may have undergone two separate WGDs: one around 69.5 Mya and the other at 3.49 Mya. This agrees with the results from earlier investigations of extensive WGD occurrences in *labiate* (Lichman et al., 2020; Sun et al., 2022). Recent research has shown that the predicted time of WGD events of *T. quinquecostatus* is highly correlated with that of *T. mandschuricus* (Ntalli et al., 2010). The divergence of Thymus species may have coincided with the occurrences of these WGD episodes.

The *Labiatae* family of plants may be found in many different regions, including thyme, lavender, mint, basil, rosemary, marjoram, sage, and skullcap plants, which possess different therapeutic properties due to their different specialized metabolites. The Lamiaceae family produces a variety of secondary metabolites, the most prevalent of which are terpenes, phenolic acids, and flavonoids (Fraternale et al., 2014). A better understanding of the evolution of plant secondary compound biosynthetic pathways will be possible with the availability of high-quality genome sequences, which will promote the development of molecular basis studies of the diversity of specialized metabolites in various members of the Lamiaceae family.

When flowers open, volatile organic compounds (VOCs) including thymol, carvacrol, p-cymene, γ-terpinene, α-terpinene, and 1,8-cineole are found in *T. mandschuricus*, more in blooms than in folige. Previous research suggested that the VOCs levels of *cistanche* bud dropped, in some cases to as low as zero as flowers opened. The levels of VOCs in bud lavender rose initially but subsequently decreased as the flowers opened (Li et al., 2019; Rao et al., 2014). Structural genes in the MEP and MVA pathways had the highest expression in *T. mandschuricus* during the initial flowering stage (FC) but then declined progressively throughout the blooming period (FS) and finally were expressed at the lowest levels during the final flowering stage (FM). This might suggest that VOCs and terpenoid synthesis gene accumulation in *T. mandschuricus* follow a similar pattern. Higher levels of expression in CHS, STS, FLS, FNS, and essential structural genes in flavonoid metabolism were also seen during the FC and FS phases, which may further imply that terpenes are produced during these blooming stages in *T. mandschuricus*. Additionally, we hypothesize that the structural genes of flavonoid metabolism of *T. mandschuricus* have expanded with a process of exceptional paralogous gene family expansion because of a high similarity between the F3’5’H-F3’H and F3H-FLS-FNS gene families.

## Conclusions

Thymus plants are often used for their taste and scent as spices, herbal teas, and insecticides. Here, we offer the genomic sequence of *T. mandschuricus*, an indigenous thyme species unique to China. The genome of *T. mandschuricus* consists of 13 pseudochromosomes, counting 587.14 Mb in size. Genomic comparisons indicated that *T. mandschuricus* was most closely related to sages like *Salvia splendens*, *Salvia bowleyana*, and *Salvia miltiorrhiza.* Ks analysis revealed two distinct WGD events in the *T. mandschuricus* genome. Key genes and regulatory networks involved in polyphenol and terpene production were identified by an in-depth investigation of the *T. mandschuricus* genome. We believe the datasets and analysis offered here will help future research in molecular breeding and functional gene discovery in Thymus.

## Materials and methods

### Sample collection

In this study, *T. mandschuricus*, an important medicinal plant, was obtained from the Maoer Mountain forest area in Shangzhi City, Heilongjiang Province, China. Young leaf samples of *T. mandschuricus* were used for genome sequencing, whereas *T. mandschuricus* collected from seven different tissues, including the initial flowering stage (FC), the final flowering stage (FM), the blooming period (FS), the tender stem (JN), the secondary branch (JE), the root (R), and the leaf (L), was used for transcriptome sequencing. It is important to note that all samples were obtained from the same strain. Three biological repeated samples were taken from each part, resulting in a total of 21 samples. The samples were immediately frozen with liquid nitrogen or carbon dioxide ice after collection and stored at −80°C. The total DNA of all samples was extracted.

### DNA and RNA extraction

Genomic DNA was extracted by classic phenol–chloroform method, whereas total RNA was extracted with a TRIzol kit. The quality and quantity of extracted DNA/RNA were assessed by an Agilent 2100 Bioanalyzer (Agilent Technologies Inc., Santa Clara, CA, USA), and integrity was evaluated on agarose gel after a stain with ethidium bromide. The resulting DNA/RNA samples were stored at −80°C until subsequent library construction and genomic/transcriptomic sequencing. All samples were collected by personnel affiliated with our research group.

### Library construction and sequencing

The libraries of *T. Mandschuricus* were constructed and sequenced on a PacBio sequencing platform (https://www.pacb.com/products-and-services/consumables/). For Hic sequencing, genomic DNA samples were pretreated according to Suhas S.P. Rao et al (Bolger et al., 2014). Under the adsorption of avidin magnetic beads, biotinized DNA was captured, and DNA fragments were end-repaired, adapter ligated, PCR-amplified, and purified in strict accordance with the Illumina Hi-C library protocol. Then, the quality of library was tested according to standard steps of library quality control. Qubit 2.0 was utilized for preliminary quantification, and the library was diluted to 1 ng/μl. Then, integrity of library DNA fragments and size of insert were detected by Agilent 2100. Then, quantitative PCR (qPCR) was employed for detecting the effective concentration of library for accurate quantification (effective concentration > 2 nM). After library inspection, different libraries were pooled according to the requirements of effective concentration and target data volume. Later, Illumina PE150 sequencing was performed according to the manufacturer’s protocols.

### Quality control of sequencing data

For the next-generation sequencing data of Illumina, low-quality reads, linker sequences, and repetitive sequences were removed by trimmomatic (Gordon and Hannon, 2010) and FASTX-Toolkit (FastQC). Finally, data quality was evaluated by fastqc 39. For long PacBio reads, mean quality for each read was calculated and only reads longer than 1 kb with a mean quality of ≥7 were retained. For the Hic data, sequences containing linkers (N > 10%) were removed using HicPro.

### Transcriptome sequencing

The rRNA was removed using the Ribo-Zero rRNA removal kit (Epicenter, Madison, WI, USA) with 1.5 μg of RNA from each sample as feedstock. The NEBNextR UltraTM Directional RNA Library Prep Kit (NEB, USA) and the NEBNextR UltraTM small RNA Sample Library Prep Kit (NEB, USA) were used according to the manufacturer’s recommendations. Transcriptome and small RNA libraries (Long noncoding RNAs (lncRNA), MicroRNAs (microRNA), and Circular RNAs (circRNA)) were constructed and sequenced on the Illumina HiSeq 2500/2000 platform. Three biological repeats were sequenced for each tissue sample.

### RNA-seq expression analysis

Clean reads were obtained after the original data were filtered, the sequencing error rate and GC content distribution were identified, and the data were then compared with the *T. mandschuricus* reference genome sequence. FPKM (fragments per kilobase of transcript per million fragments) values were used to indicate transcript or gene expression levels. The original count data were analyzed by using DESeq2 v1.22.1 software. The Benjamini–Hochberg method was employed to adjust the probability (P-value) of the hypothesis test in order to obtain the false discovery rate (FDR). The differentially expressed genes were selected as screening criteria, occurring one or more times, with FDR < 0.05. Enrichment analysis was conducted using the Kyoto Encyclopedia of Genes and Genomes (KEGG), and a hypergeometric distribution test was used with pathway units.

### Estimation of genomic size and genomic assembly

Genomic sizes were estimated by k-mer method with short-insert library reads (Koren et al., 2017). Here, 17-mer was selected for k-mer analysis and genomic size (Mb) estimated with the following formula: G = Knum/Kdepth, where Knum and Kdepth denoted total number and peak depth of 17-mers, respectively. *De novo* assembly of the long reads from the PacBio SMRT Sequencer was performed using wtdbg2 (Luo et al., 2012). The Fuzzy Bruijn Graph algorithm was used to assemble and integrate 1,024-bp sequences from the reads into vertex sequences, and, then, based on the position of the vertex sequences on the reads, the vertex sequences were concatenated to obtain the genome sequences. Subsequently, the Hi-C sequencing data were aligned to the assembled scaffolds by Burrows–Wheeler aligner-maximum exact matches (BWA-MEM) (Vasimuddin et al.,), and the scaffolds were clustered onto chromosomes with LACHESIS (Kajitani et al., 2014).

Quality assessment of genomic assemblies for *T. mandschuricus* was performed by BUSCO and CEGMA (Parra et al., 2007). BUSCO (Benchmarking Universal Single-Copy Orthologs) utilized a single-copy orthologous gene library along with tblastn, Augustus, HMMER, and other software tools to evaluate the integrity of assembled genomes. CEGMA (Core Eukaryotic Genes Mapping Approach) was adopted for selecting conserved genes (458 genes) in six eukaryotic model organisms and constructing a core gene library along with tblastn, genewise, and geneid software tools, which were conducted to evaluate the integrity of assembled genome.

### Genomic annotation

Genome annotation comprises three main aspects: repeat sequence annotation, gene annotation, and non-coding RNA annotation. Two methods, namely, homologous sequence alignment and *de novo* prediction, were employed for repeat sequence labeling. The homologous sequence alignment method utilized Repeatmasker and repeatproteinmask software to identify the repeated sequences. *Ab initio* prediction was carried out using software such as LTR_FINDER, RepeatScout, RepeatModeler, and others. The ab repeat sequence library was established first, followed by prediction using Repeatmasker software (Chen, 2004; Flynn et al., 2020; Price et al., 2005; Xu and Wang, 2007). Protein-coding genes were annotated using a combination of *de novo* prediction, homology-based annotation, and transcription-based repetitive hidden genome annotation. For *ab initio* forecasting, Augustus (v.3.2.1) and GENSCAN (v.1.0) were used (Stanke and Morgenstern, 2005). The protein sequences of related species were downloaded from the NCBI database based on homology annotations. BLAST (Altschul et al., 1990) and GeneWise (Birney et al., 2004) were then used to predict the genetic structure. The annotation of gene sets and databases such as SwissProt (http://www.uniprot.org/), Nr (http://www.ncbi.nlm.nih.gov/protein), Pfam (http://pfam.xfam.org), KEGG (http://www.genome.jp/kegg/), InterPro (https://www.ebi.ac.uk/interpro/), and others were compared for gene function information. tRNAscan-SE software was used to locate tRNA sequences in the genome. INFERNAL software was employed to predict miRNA and snRNA sequence information in Rfam using the Rfam family covariance model.

### Identification of orthologous genes and phylogenetic tree construction

Homologous species and nine closely related species were identified. The similarity relationship between the protein sequences of each species was determined using the whole-to-whole-embryo method, and the results were clustered using OrthoMCL software (Li et al., 2003). A maximum likelihood phylogenetic tree was constructed based on multiple sequence alignments using RAxML (Stamatakis, 2014).

### Estimation of gene family expansion

Expansion and contraction of gene family was determined by CAFÉ software (v.3.1) (De Bie et al., 2006). Phylogenetic tree and divergence time of previous steps were imported into CAFE to infer the changes of gene family sizes using a probability model. Protein sequences that have been annotated as being involved in the terpenoid backbone biosynthesis pathway have been retrieved from all plant species using the KEGG database (Ko00900). These sequences include ACAT, HMGS, HMGR, MVK, PMK, MVD, DXR, DXS, MCT, CMK, MDS, HDS, HDR, IDI, and generate geranyl diphosphate synthase [(FPPS)/GGPPPS/FPPS]. Then, using BLASTP and setting the threshold for the E-value to 1e-5, we searched for proteins that were similar to those in the genome of *T. mandschuricus*. We predicted the TPS genes by using a BLAST method that was based on conserved domains (PF01397 and PF03936) and a homolog-based algorithm. Conserved domains were used as search queries inside HMMER’s hmmsearch module (Johnson et al., 2010). To identify the TPSs of *T. mandschuricus*, TPS protein sequences from all plant species were used as queries. For the purpose of BAHD identification, members of the BAHD family from Atha were used as queries for the BLASTP (1e-5) prediction of *T. mandschuricus* BAHD. We used the CYP450 protein sequences of Ath as queries to look for homologs and conserved domains in order to locate the genes that code for the CYP450 proteins (PF00067).

## Data availability statement

The datasets presented in this study can be found in online repositories. The names of the repository/repositories and accession number(s) can be found below: https://www.ncbi.nlm.nih.gov/, PRJNA929318.

## Author contributions

LJ: Data curation, Formal Analysis, Investigation, Visualization, Writing – original draft, Writing – review & editing. NX: Investigation, Visualization, Writing – original draft. BX: Investigation, Visualization, Writing – review & editing. WG: Formal Analysis, Investigation, Resources, Writing – review & editing. QM: Formal Analysis, Investigation, Resources, Writing – review & editing. QL: Formal Analysis, Investigation, Resources, Writing – review & editing. YS: Formal Analysis, Investigation, Resources, Writing – review & editing. SX: Formal Analysis, Investigation, Resources, Writing – review & editing, Conceptualization, Data curation, Funding acquisition, Methodology, Project administration, Software, Supervision, Validation, Visualization. MH: Conceptualization, Supervision, Writing – review & editing. HG: Conceptualization, Funding acquisition, Project administration, Resources, Supervision, Writing – review & editing.
