# Supplementary material for: Chromosome-level genome of Thymus mandschuricus reveals molecular mechanism of aroma compounds biosynthesis
Source: Front Plant Sci. 2024 Mar 13;15:1368869. doi: 10.3389/fpls.2024.1368869 (PMC10965588; doi:10.3389/fpls.2024.1368869)
Supplement: Supplementary file 1 [file DataSheet_1.pdf]

Table.S1 Summary of the *T. mandschuricus* genome

| Parameter | Size or number |
|---|---|
| Estimate of genome size (survey), Mb | 528.75 |
| Assembled genome size, Mb | 587.05 |
| Chromosome-anchored scaffolds, Mb | 531.02 |
| GC content, % | 40.13 |
| Complete CEGMA, % | 95.56 |
| Complete BUSCOs, % | 96.80 |
| Repeat density, % | 67.70 |
| Long terminal repeat (LTR) density, % | 54.97 |
| Number of protrin-coding genes | 30,428 |
| Number of annotated genes | 29,343 |
| Number of rRNA | 2,198 |
| Number of tRNA | 856 |
| Number of miRNAs | 4,474 |
| Number of snRNAs | 2,106 |
