# Supplementary figures and images for: Chromosome-level genome of Thymus mandschuricus reveals molecular mechanism of aroma compounds biosynthesis
Source: Front Plant Sci. 2024 Mar 13;15:1368869. doi: 10.3389/fpls.2024.1368869 (PMC10965588; doi:10.3389/fpls.2024.1368869)

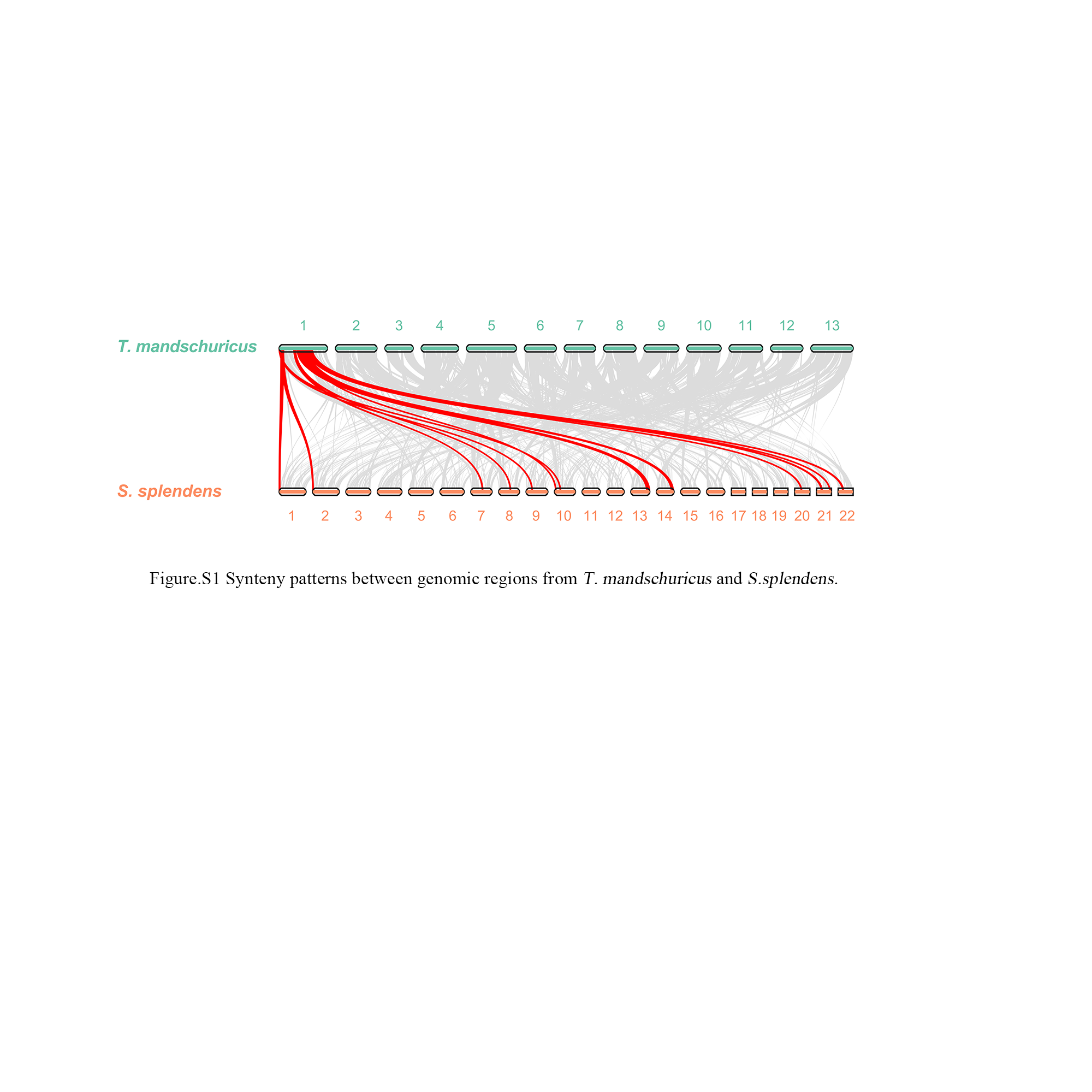

Supplement: Supplementary file 2 [file Image_1.tif]
